# Biomaterial-Driven Modulation of Macrophage Polarization in an Experimental Environment of Implant-Associated Infection Mediated by *Staphylococcus aureus*: A Systematic Review of Preclinical Studies

**DOI:** 10.3390/jfb17070342

**Published:** 2026-07-14

**Authors:** Giorgia Codispoti, Maria Sartori, Michael Salvatore, Andrea Liberatore, Liliana Gabrielli, Tiziana Lazzarotto, Gianluca Giavaresi

**Affiliations:** 1 Surgical Sciences and Technologies, IRCCS Istituto Ortopedico Rizzoli, Via di Barbiano, 1/10, 40136 Bologna, Italy; giorgia.codispoti@ior.it (G.C.); michael.salvatore@ior.it (M.S.); gianluca.giavaresi@ior.it (G.G.); 2 Microbiology Unit, IRCCS Azienda Ospedaliero-Universitaria di Bologna, Via Massarenti 9, 40138 Bologna, Italy; andrea.liberatore@studio.unibo.it (A.L.); liliana.gabrielli@aosp.bo.it (L.G.); tiziana.lazzarotto@unibo.it (T.L.); 3 Department of Medical and Surgical Sciences, Alma Mater Studiorum, University of Bologna, Via Massarenti 9, 40138 Bologna, Italy

**Keywords:** biomaterials, implant-associated infection, *Staphylococcus aureus*, macrophage polarization

## Abstract

Implant-associated infections (IAIs) caused by *Staphylococcus aureus* are a major cause of orthopedic implant failure, driven by biofilm formation and chronic inflammation. Macrophages regulate bacterial clearance and tissue repair through polarization into pro-inflammatory (M1) and anti-inflammatory (M2) phenotypes. This systematic review evaluated preclinical in vivo studies according to PICO criteria (Population: IAI animal models; Intervention: functionalized biomaterials; Comparator: non-functionalized controls; Outcome: bacterial burden reduction associated with macrophage reprogramming). A PRISMA-guided search of PubMed, Scopus, Web of Science, and Embase (January 2015–December 2025) identified 23 eligible studies, assessed using SYRCLE’s risk-of-bias tool. Due to heterogeneity, a narrative synthesis was performed without meta-analysis. Effect measures included bacterial CFU reduction, macrophage polarization markers, cytokine expression, and histological outcomes. In 87.5% of studies, macrophage modulation was associated with reduced bacterial load, biofilm disruption, and improved peri-implant tissue repair and bone integration. These findings support immunomodulatory biomaterials as promising strategies to manage IAIs through combined antibacterial and immune-regulatory mechanisms. However, further validation in larger animal models is required, particularly for nanomaterials. The protocol of this systematic review was registered on the Open Science Framework.

## 1. Introduction

In the orthopedic field, the use of devices (e.g., prostheses, nails, cages, screws) to treat pathological conditions affecting musculoskeletal tissues represents a standard and consolidated clinical practice from which patients generally benefit [1,2]. Despite generally favorable outcomes, material-related risks and adverse events may lead to significant clinical and psychosocial consequences for patients [3].

The most serious complication is the susceptibility of prosthetic implants to bacteria colonization, which may evolve into implant-associated infections (IAIs). IAIs affect approximately 1.5–2.5% of primary total hip and knee arthroplasty [4,5], and are associated with considerable morbidity, chronic pain and functional impairment, and in the worst cases, the need for complex revision procedures [6]. Revision surgery further increases the risk of reinfection, with IAI incidence reaching up to 20% [7], imposing a significant burden both for patients and healthcare systems as implant numbers continue to rise [8,9,10].

In the IAI field, extensive research focuses on the mechanisms leading to bacterial adhesion, colonization and subsequent biofilm formation on devices’ surfaces [11]. Gram-positive bacteria predominate, with *Staphylococcus aureus* representing one of the main causative agents. Bacterial attachment is mediated by microbial adhesive components that recognize host matrix proteins such as fibronectin, fibrinogen, and vitronectin, initiating rapid surface colonization and biofilm development through stages of attachment, maturation, and dispersal of planktonic cells [12]. Biofilms confer high tolerance to antimicrobial agents by limiting drug penetration and shielding bacteria from immune responses [13]. In addition, biofilm-forming pathogens such as *S. aureus* employ multiple immune evasion strategies, including toxin production, inhibition of opsonization and phagocytosis, interference with complement activation, and intracellular survival within macrophages, all contributing to infection persistence [14,15].

In this dynamic microecosystem located at the implant–host interface, innate and adaptive immune responses play crucial roles [16].

Among innate immune cells, tissue-resident macrophages are abundant and rapidly activated responders to implanted materials that critically influence implant outcomes. They clear debris and pathogens, regulate the foreign body response, and support tissue integration, but in the presence of infection they may also sustain inflammation and impair tissue repair [14]. Upon reaching the site of implant, macrophages exhibit remarkable functional plasticity, adapting their phenotype to local microenvironmental cues through a process known as polarization [17]. This phenomenon is commonly described by the M1 pro-inflammatory phenotype, induced by interferon-gamma (IFN-γ) and lipopolysaccharide (LPS), and characterized by microbicidal activity, reactive oxygen species (ROS) and pro-inflammatory cytokine production, such as tumor necrosis factor-alpha (TNF-α), interleukin-1 beta (IL-1β) and interleukin-6 (IL-6). On the other hand, the M2 anti-inflammatory phenotype is usually driven by interleukin-4 (IL-4) and interleukin-13 (IL-13), and it is associated with immune regulation, tissue remodeling and production of interleukin-10 (IL-10) and transforming growth factor-beta (TGF-β) [18]. Although simplified, this framework is valuable for understanding macrophage behavior in IAIs.

During the early stages, planktonic bacteria activate macrophages via Toll-like receptor 2 (TLR2), driving an M1 pro-inflammatory response aimed at bacterial clearance [14]. As a biofilm matures, a shift towards the M2 anti-inflammatory phenotype is promoted by immune evasion and S. aureus-derived factors, supporting bacterial persistence and chronic infection establishment [19,20,21].

Dysregulated macrophage polarization at the implant site may lead to pathological outcomes: excessive M1 activation promotes osteoclastogenesis [22] and peri-implant bone resorption, contributing to osteolysis and implant failure [23], whereas sustained M2 response may create a permissive niche for chronic bacterial persistence [24]. Together, these alterations identify macrophage polarization as a critical pathological node in IAI.

Current treatment strategies for IAI rely mainly on systemic antibiotic therapy combined with surgical debridement or implant removal in the worst cases. However, the biofilm microenvironment markedly limits the efficacy of conventional antibiotics, by hindering drug penetration and decreasing bacterial susceptibility [25]. Consequently, complete eradication of biofilm-associated infections remains difficult to achieve with systemic approaches alone [26].

This therapeutic gap highlights the need for alternative strategies capable of both preventing bacterial adhesion [27] and modulating the host immune response through macrophage polarization [28]. Therefore, this review aims to evaluate in vivo biomaterial-based approaches designed to modulate macrophage polarization as a novel strategy for the prevention and treatment of *S. aureus* implant-associated infections.

## 2. Materials and Methods

### 2.1. Search Strategy

This literature review was performed through a systematic search across four electronic databases (PubMed^®^, Scopus^®^, Web of Science^®^ and Embase^®^: www.pubmed.gov, www.scopus.com, www.webofscience.com and www.embase.com), according to the PRISMA guidelines (Supplementary Table S1) [29]. The review protocol was registered on the Open Science Framework (OSF: https://osf.io/ur9q5; accessed on 13 July 2026)). The search strategy included following keywords combined with booleans: “Staphylococcus Aureus” OR “S. Aureus” AND “implant-associated infection” OR “periprosthetic infection” OR “peri-implant infection” OR “IAI” OR “peri-prosthetic joint infection” OR “PJI” AND “macrophage.” The full search strategies for each database are reported in Table 1. Screening and analysis of the retrieved records were carried out in a blinded and independent manner by two reviewers (G.C. and MA.S.). A web-based tool for narrative and systematic reviews, Rayyan [30], was used for the detection and removal of duplicates and the determination of the studies included or excluded based on titles and abstracts.

The inclusion criteria comprised all in vivo preclinical studies investigating whether a new generation of biomaterials tested in the context of implant-associated infection (IAI) sustained by S. aureus can also affect macrophage repolarization. Additional criteria included publication in English within the timeframe of 1 January 2015 to 31 December 2025. Exclusions criteria consisted of papers written in languages other than English, reviews, abstracts, clinical studies, editorials, comments, publisher’s notes, white papers, and letters. Studies lacking the assessment of *S. aureus* exposure or an implant-associated infection model were also excluded. The reference list of the included studies was manually screened in order to identify further relevant papers not captured by the electronic database search. Discrepancies between reviewers were resolved through discussion and, when needed, with the involvement of a third reviewer (G.G.).

### 2.2. Data Extraction

In accordance with PRISMA suggestions, a well-defined PICO question was formulated to facilitate accurate data extraction from included studies: Population (P): animal models of IAI caused by S. aureus, Intervention (I): functionalized biomaterials or approach in which macrophage polarization at the implant site was investigated, Comparator (C): non-functionalized implants, or control groups receiving implants without functionalization, Outcome (O): reduction in bacterial burden associated with macrophage reprogramming. Starting from the study focus, G.C. and MI.S. systematically extracted the key characteristics from the selected studies. The collected data from in vivo studies were: the type, size and characteristics of the tested material/approach; specifics of the animal model used for implant-associated infection (IAI), including species, strain, sex, age, number of animals, and experimental groups; the implantation site for the IAI model; the microbiological investigations to evaluate the bacterial load and biofilm assessment; the tests used for macrophage reprogramming assessment; the established experimental times; the main findings; and, if reported, the signaling pathways analyzed.

A third author (MA.S.) checked the extracted data for accuracy and completeness. Any disagreements were resolved through discussion and, when necessary, with the consultation of another reviewer (G.G.). Extracted data are reported in Table 2.

### 2.3. Risk-of-Bias Assessment

The SYRCLE tool [53], based on a 10-domain checklist including “Selection,” “Performance,” “Detection,” “Attrition,” “Reporting” and other bias, was used to assess the risk of bias of the in vivo studies. The risk of bias was classified as low, high, or unclear, depending on whether the domains were clearly reported, not reported, or reported ambiguously. Two authors (G.C. and MA.S.) conducted the assessment independently, and disagreements were resolved through discussion or by consulting a third reviewer (MI.S.).

### 2.4. Data Synthesis

Results for each outcome were reported using the original data from the included studies, including the CFU counts, percentage of bacterial load reduction, and qualitative or semi-quantitative microbiological, histological and macrophage polarization assessments. Due to heterogeneity in the study design, biomaterials, and outcome measures, a quantitative meta-analysis was not performed, and the results were synthesized descriptively. No sensitivity analyses or assessment of the certainty of the evidence were conducted. No assessment of risk of bias due to missing results in the synthesis was performed.

## 3. Results

### 3.1. Search Strategy

The review process began with a comprehensive literature search using the predefined search strings. This search identified 61 articles from PubMed (www.pubmed.gov), 33 from Web of Science (www.webofscience.com), 74 from Scopus (www.scopus.com) and 871 from Embase (www.embase.com).

All retrieved references were imported into Rayyan, a publicly available software tool for systematic review management, to remove duplicates [30]. This step led to the exclusion of 141 duplicate records, leaving 898 articles for screening.

**Figure 1:**
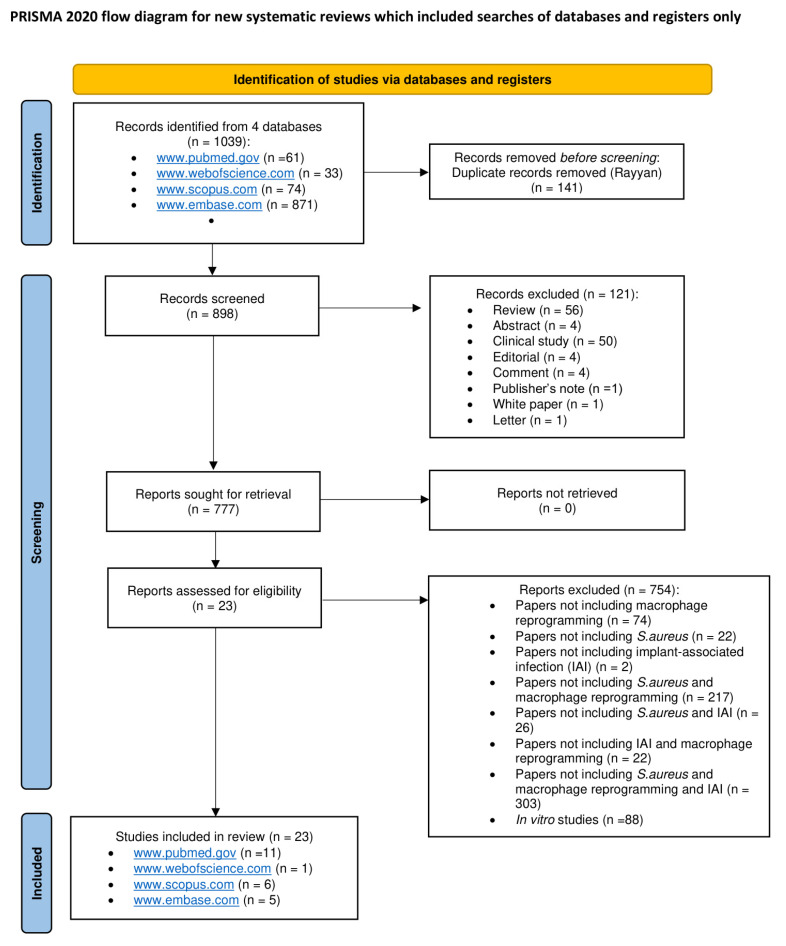
Search workflow. Search strategy according to the PRISMA guidelines [29].

During the initial title and abstract screening, 121 records were excluded: 56 reviews, 4 abstracts, 50 clinical studies, 4 editorials, 4 comments, 1 publisher’s note, 1 white paper and 1 letter. The remaining 777 articles underwent full-text screening, which resulted in the exclusion of 754 non-inherent studies, as illustrated in the PRISMA flow chart (Figure 1). Following this selection process, 23 in vivo studies met the eligibility criteria and were included in the review (Figure 1): 11 from PubMed, 1 from Web of Science, 6 from Scopus and 5 from Embase. Manual screening of the reference lists of all included studies did not yield additional eligible records.

### 3.2. Risk-of-Bias Assessment

**Figure 2:**
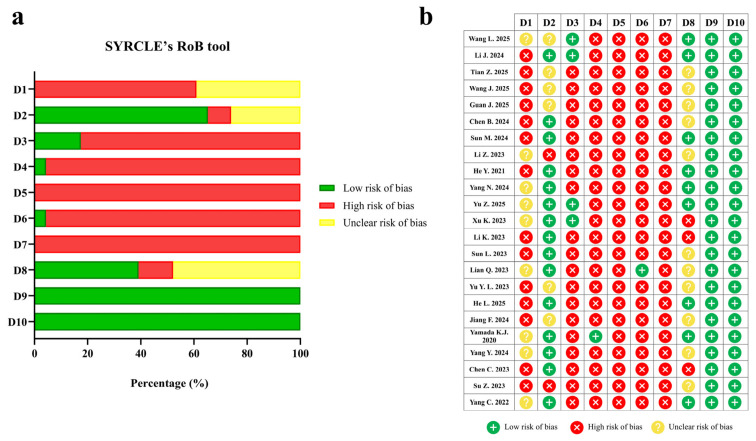
In vivo risk of bias results. Overall risk of bias (**a**) and assessment for each in vivo study (**b**) by applying SYRCLE’s RoB tool [31]. Studies are presented in the same order as in Table 2 (functionalized PEEK implants, functionalized titanium implants, and nanomaterials) [20,32,33,34,35,36,37,38,39,40,41,42,43,44,45,46,47,48,49,50,51,52,53]. Each domain (D; D1—sequence generation, D2—baseline characteristics, D3—allocation concealment, D4—random housing, D5—blinding of caregivers, D6—random outcome assessment, D7—blinding of outcome assessment, D8—incomplete outcome data, D9—selective outcome reporting and D10—other bias) is reported as low risk (green), high risk (red) and unclear risk (yellow) of bias.

The risk-of-bias assessment revealed that most domains were assessed as “high risk,” as illustrated in Figure 2a,b. Domain 1, “sequence generation,” was at high risk in over half of the studies (60.9%) due to a lack of reporting on the randomization process for animal allocation. Similarly, domain 3, “allocation concealment,” was rated high risk in the majority of papers (82.6%), as the characteristics used for group assignment were not fully described, and no study clearly explained how allocation was performed. Domains 4, “random housing,” and 6, “random outcome assessment,” showed a high risk of bias in 95.7% of studies, as 22 out of 23 studies did not specify how animals were housed within the facility after group assignment, nor whether experimental samples were analyzed in random order. Domains 5 (“blinding” as performance bias) and 7 (“blinding” as detection bias) were high risk in all studies (100%), because investigators were aware of group assignments and assessors were not blinded during outcome evaluation. Finally, Domain 8, “incomplete outcome data,” was rated as unclear risk in 47.8% of studies, due to discrepancies between the number of animals reported in the methods and those reported in the results.

### 3.3. In Vivo Preclinical Studies

The in vivo studies were grouped according to the type of material investigated and the treatment performed to confer antibacterial properties: 2 studies out of 23 (8.7%) developed functionalized polyetheretherketone (PEEK) implants [31,32], 14 papers out of 23 (60.9%) investigated functionalized titanium implants [33,34,35,36,37,38,39,40,41,42,43,44,45,46], while 7 out of 23 (30.4%) adopted nanomaterials [20,47,48,49,50,51,52] for the treatment of implant-associated infections.

#### 3.3.1. Functionalized PEEK Implants

**Figure 3:**
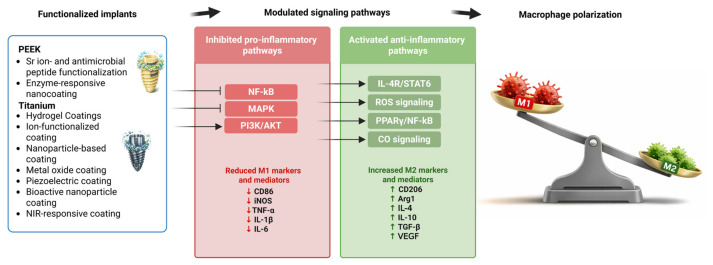
Modulation of macrophage polarization by functionalized implants. Implant surface modifications modulated intracellular pathways, either inhibiting pro-inflammatory pathways (e.g., NF-κB and MAPK), resulting in decreased (↓) expression of M1 markers (e.g., CD86 and iNOS) and pro-inflammatory cytokines (e.g., TNF-α, IL-6 and IL-1β), or promoting signaling pathways associated with M2 polarization and tissue repair (e.g., PI3K/AKT and related downstream mediators like PPARγ), leading to increased (↑) expression of M2 markers (e.g., CD206, ARG1, IL-4, IL-10, VEGF and TGFβ).

In the study by Wang et al. [31], a PEEK implant was modified with a polydopamine coating, followed by the deposition of strontium (Sr) ions and the antimicrobial peptide PMAP-36 (PEEK-PDA-Sr/AMP). In Li et al.’s study [32], a PEEK implant was firstly sulfonated to produce SPEEK. This sulfonated PEEK was then functionalized with glucose-gated nanocoating, which contained glucose oxidase (GOx) and manganese carbonyl nanocrystals (MnCO), developing a near-infrared (NIR)-responsive system. In both studies, a male Sprague–Dawley rat was selected as the animal model, in which femoral and tibial defects were surgically created and injected with an S. Aureus suspension to induce IAI. The bacterial suspension concentration was only reported in the Wang et al. study, which used a bacterial inoculum of 10^4^ CFU/mL. Macrophage reprogramming was assessed using multiple techniques, including immunohistochemistry (IHC) focused on the expression of M1 and M2 markers (cluster of differentiation, CD86 and CD206) in the peri-implant tissue, immunofluorescence, enzyme-linked immunosorbent assay (ELISA) and real-time quantitative polymerase chain reaction (RT-qPCR) to evaluate the expression of surface and intracellular markers of M1 and M2 macrophages (inducible nitric oxide synthase, iNOS, and CD206), as well as the expression of pro-inflammatory cytokines (IL-6 and TNF-α). Both studies showed an increase in CD206 expression, an M2 marker, following treatment, as well as a reduction in the intracellular M1 marker iNOS and the pro-inflammatory cytokines secreted by them. Macrophage reprogramming after these treatments was supported by inhibition of the pro-inflammatory nuclear factor kappa-light-chain-enhancer of activated B cells (NF-kB) pathway, mediated by the release of Sr ions in Wang’s study and by hyperglycemia in Li’s study, by activating glucose oxidase, which promotes the release of carbon monoxide (Figure 3). These changes in macrophage phenotype impacted the infection outcomes. In Wang’s study, the treatment with PEEK-PDA-Sr/AMP completely cleared the implantation site from bacteria, as revealed by the absence of bacterial colonies in peri-implant tissue sections stained with Giemsa. The antimicrobial peptide activity combined with Sr-mediated immunomodulation demonstrated that the functionalized PEEK implant can effectively control the infection. Similarly, SPEEK-GOx/MnCO treatment combined with photodynamic therapy (PDT) significantly reduced bacterial load and viability in Li’s approach. Under NIR, the decomposition of MnCO was accelerated, promoting the increased local release of carbon monoxide (CO), which acted as a gas therapeutic agent contributing to the antibacterial activity. This effect was associated with macrophage metabolic reprogramming toward the anti-inflammatory but bactericidal state.

However, despite the promising infection-related outcomes, no quantitative microbiological data confirming bacterial clearance, such as colony forming unit (CFU) count, were provided. In Wang’s study, only the initial inoculum concentration was reported, whereas Li’s study did not specify the bacterial load used to test the biomaterial.

Nevertheless, histological observations on peri-implant tissue sections stained with Masson’s trichrome suggested that functionalized PEEK implants promoted peri-implant matrix deposition and new bone formation, in line with immunohistochemistry results of osteogenic markers, runt-related transcription factor 2 (RUNX2), osteocalcin (OCN) and Collagen Type I Alpha 1 Chain (COL1A1).

#### 3.3.2. Functionalized Titanium Implants

Titanium-based substrates have been functionalized by adopting different strategies. One approach involved the deposition of piezoelectric materials such as barium titanate (BaTiO3) [37,43] and polyvinylidene fluoride (PVDF) [44], which respond to ultrasound and promote macrophage reprogramming through ROS generation or direct interaction with macrophages. Surface modifications also included interfacial functionalization based on nanoparticles (NPs) such as polydopamine (PDA) [46] and iron (III)–tannic acid complex (FeIIITA) NPs [42] conjugated to antibacterial agents and responsive to NIR, which produced photothermal effects for bacteria killing. Other emerging approaches included hybrid functionalization, in which pure titanium was coated with a composite system combining inorganic components such as metallic ions or a metal oxide like zinc oxide (ZnO) [36] with organic materials such as amyloid lactoferrin and poly-γ-glutamic acid (γ-PGA) [34,41], or with hybrid coatings, including gelatin methacryloyl (GelMa) hydrogel [35,39], which served as delivery platforms for bioactive molecules [40] and antibacterial drugs [33,45]. Finally, Li et al. investigated a bioengineered strategy, including nanostructured layers linked to peptide NPs carrying a DNA plasmid coding for macrophage Chimeric Antigen Receptor (CAR) and allowing for specific recognition of S. Aureus [38]. Rats were used in the majority of studies (12 out of 14, 85.7%) [33,34,35,36,37,40,41,42,43,44,45,46], most of which were male, while the remaining studies employed male mice (2 out of 14, 14.3%) [38,39] to establish an IAI model. Titanium-based implants were predominantly placed in the femoral or tibial intramedullary canal or implanted subcutaneously, reflecting their intended orthopedic applications. In contrast, in the study by Chen et al. [36], the material was implanted in the maxilla to simulate dental application. To induce infection, selected strains of S. aureus or Methicillin-Resistant S. Aureus (MRSA) were introduced directly into the created bone defect in 8 of 14 studies (57.1%) [33,34,35,38,39,41,42,45], whereas in 3 studies materials were pre-exposed to bacterial suspension prior to the implantation (21.4%) [36,37,40], or implanted after pre-formed biofilm on the titanium surface (3 out of 14, 21.4%) [43,44,46]. Bacterial concentrations ranged from 1 × 10^4^ to 1 × 10^8^ CFU/mL.

Macrophage reprogramming was assessed by multiple techniques, including surface marker analysis by flow cytometry, as well as gene and protein expression and tissue-level evaluation through RT-qPCR, ELISA, immunohistochemistry, and immunofluorescence. Markers characteristic of M1 macrophages (CD86, CD80, iNOS) and M2 macrophages (arginase 1, Arg-1, and CD206), as well as pro-inflammatory cytokines (TNF-α, IL-6 and IL-1β) and anti-inflammatory cytokines (IL-10, and TGF-β), were predominantly analyzed in peri-implant tissues at experimental times ranging widely from 4 h to 8 weeks post IAI establishment.

Overall, 64.3% of the included studies (9 out of 14) [33,34,35,36,39,40,41,44,46] reported a shift toward an anti-inflammatory macrophage phenotype following material-based treatments, as evidenced by increased expression of M2-associated markers such as CD206 and Arg-1, together with a concomitant reduction in M1 markers and pro-inflammatory cytokines. This immunomodulatory effect induced by treatments was generally associated with a significant reduction in bacterial CFUs, and fewer histologically detectable bacteria revealed with hematoxylin & eosin (H&E), Giemsa and/or Gram staining. These findings were further supported by pathway analyses, which revealed that M2 polarization was promoted through multiple mechanisms, including (i) inhibition of inflammatory signaling pathways involved in M1 differentiation, such as NF-κB [33,40,46]; (ii) material-driven increases in ROS; (iii) release of immunomodulatory factors, including IL-4 [39], known to favor M2 polarization; (iv) release of bioactive ions such as silver ions (Ag^+^) to switch macrophages to the M2 type [41]; and (v) the generation of self-stimulated electric fields capable of regulating macrophages toward an anti-inflammatory phenotype [44]. Notably, in the studies by Wang [34], Guan [35], and Chen [36], the signaling pathways underlying macrophage polarization were not specifically investigated. By contrast, 35.7% of the studies (5 out of 14) [37,38,42,43,45] revealed a predominantly pro-inflammatory response by macrophages, characterized by elevated expression of M1-associated surface and intracellular markers (CD86 and iNOS) and increased production of key pro-inflammatory cytokines (TNF-α and IL-1β). The generation of a robust pro-inflammatory response by functionalized titanium implants facilitated bacterial clearance, with significantly decreased CFU counts or bacteria having been eradicated; suppressed biofilm, as evidenced by Scanning Electron Microscopy (SEM); and a lower inflammatory reaction surrounding the IAI site, as shown by histological analyses.

Mechanistic analyses indicated that M1 polarization was promoted by increased production of reactive oxygen and nitrogen species (ROS and NO) [37], activation of pro-inflammatory signaling cascades such as phosphoinositide 3-kinase–protein kinase B (PI3K–AKT), mitogen-activated protein kinase (MAPK) [43], interleukin-12 Receptor–Signal Transducer and Activator of Transcription 4 (IL12R–STAT4) and Interferon-gamma–Signal Transducer and Activator of Transcription 1 (IFN-γ–STAT1), with the latter being driven by IL-12 delivery [45], as depicted in Figure 3. Other factors include the engagement of TLRs by surface-functionalizing molecules [42], and the expression of specific macrophage receptors that boost the bactericidal activity against S. aureus [38]. M2 polarization, particularly in studies involving titanium implants, was associated with enhanced peri-implant matrix deposition and bone formation. This was supported by micro-CT analyses showing improvements in bone parameters (trabecular number, Tb.N, trabecular thickness, Tb.Th, bone mineral density, BMD, bone volume–total volume ratio, BV/TV), as well as by histological stainings such as Masson’s trichrome, which highlighted new collagen fiber deposition and osteoid tissue formation. These findings were further confirmed by immunohistochemistry and immunofluorescence analyses of osteogenic markers, including RUNX2, alkaline phosphatase (ALP), OCN and osteopontin (OPN) in peri-implant tissue, which reflect active osteogenesis and bone formation.

#### 3.3.3. Nanomaterials

Several studies have investigated nanomaterials as therapeutic platforms for IAI treatment. In particular, four studies employed physically activated nanomaterials, with three out of four investigating NIR-responsive nanostructures consisting of organic materials, hybrid materials or organic–inorganic metal–organic frameworks (MOFs) to exploit the photothermal effect to penetrate the biofilm and stimulate ROS production through copper ion (Cu+) release. One study proposed a white light-responsive nanoplatform, based on organic–inorganic materials, in which a photocatalyst was used to boost ROS production and enhance immune response against MRSA [48]. Other approaches included the use of intrinsically active MOF [49], represented by organic nanoparticles delivering a metabolic inhibitor for macrophage polarization modulation [20], and inorganic metal and non-metal nanosheets stabilized by polyvinylpyrrolidone (PVP) [52]. Finally, controlling the delivery of drugs such as rosiglitazone for peri-implant microenvironment modulation represents another exploited strategy [50].

All studies used mice (7 out of 7, 100%), principally male, in which the materials were placed either subcutaneously (5 out of 7, 71.4%) [47,48,50,51,52] or in surgically created femoral defects (2 out of 7, 28.6%) [20,49]. Implant-associated infection was induced by pre-exposure of the implants to S. aureus or MRSA suspensions [20,50,51], implantation of pre-formed biofilms [47], or direct injection of bacterial suspensions into the defect site after implantation [48,49,52], with reported concentration ranging from 10^3^ to 10^7^ CFU/mL, although this information was not consistently specified across all studies. In most studies, nanomaterial-based treatments were administered as single injections at the implantation site between 1 and 3 days after IAI induction in the animal model.

Macrophage reprogramming was evaluated using methods similar to those applied for titanium-functionalized implants. Surface and intracellular markers for M1-type (CD86, iNOS) and M2-type (CD206, Arg-1) macrophages were analyzed. Pro-inflammatory (TNF-α, IL-6, IL-1β, hypoxia-inducible factor-1 (HIF-1α), IL-12 and triggering receptor expressed on myeloid cells 1 (Trem1)) and anti-inflammatory (IL-10, Vascular and Endothelial growth factor (VEGF) Trem2) factors were evaluated. These parameters were assessed at the gene and protein levels, as well as at the tissue level, using flow cytometry, RT-Qpcr, ELISA and immunofluorescence. This occurred within a time frame ranging from 3 days to 2 weeks after surgery.

**Figure 4:**
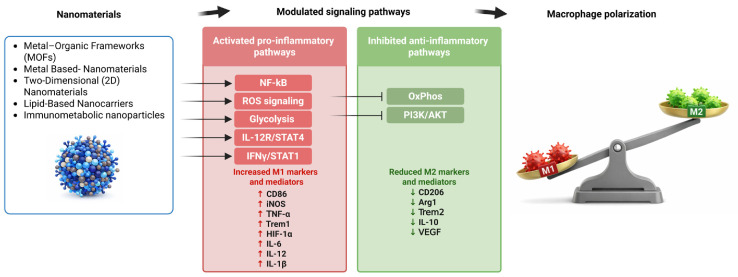
Modulation of macrophage polarization by nanomaterials. Various nanomaterials activated pro-inflammatory signaling cascades (e.g., NF-kB, ROS) or inhibited anti-inflammatory pathways (e.g., PI3K/AKT and related downstream mediators, OxPhos), leading to upregulation (↑) of M1-associated markers and mediators (CD86, iNOS, TNF-α, HIF-1α, IL-6, IL-12, IL-1β) and downregulation (↓) of M2-linked ones (CD206, Arg1, Trem2, IL-10, VEGF). Collectively, nanomaterials shifted the macrophage phenotype balance toward M1 polarization.

Except for Chen et al. [50], all works reported a significant shift in macrophage populations toward a pro-inflammatory phenotype, sustained by increased ROS production, pathogen-associated molecular patterns (PAMPs) recognition and metabolic reprogramming toward a glycolytic profile (Figure 4). This was correlated with bacterial killing and biofilm disruption, as observed through CFU counts, SEM analyses and histological stainings, evidencing their antibacterial effects mediated by immunomodulation. Jiang’s group observed an early increase in M1 type during infection, followed by a later transition toward M2 phenotype, promoting a reduction in bacterial burden, as assessed by the measurement of infection area size, CFU count and biofilm observation by SEM, and providing support to tissue repair and healing [48]. On the other hand, the approach proposed by Chen promoted M2 polarization through rosiglitazone-mediated activation of PPAR-γ signaling, which inhibited Nf-kB activity, the main molecular master orchestrating M1 polarization and inflammatory cytokine production. However, the study did not provide clear in vivo evidence of complete bacterial eradication mediated by M2 type activated by the treatment. Nevertheless, preliminary in vitro experiments demonstrated that indocyanine green + rosiglitazone (ICG + RSG) NPs were able to restore phagocytic activity of macrophages against pathogens, destroying biofilm and inhibiting bacterial growth by employing photothermal and photodynamic therapies [50].

## 4. Discussion

In the current scientific landscape, research on IAI has mainly focused on the development of antibacterial materials aimed at preventing, reducing and/or counteracting bacterial adhesion and growth onto material surfaces. Increasing attention is now being paid to the comprehension of the role exerted by the immune system as well, which is known to be actively involved in infection events. Consequently, the next-generation materials are not only designed to exert antimicrobial action but are also engineered to interact with and stimulate the immune cell response, especially macrophages [54], which are key regulators of inflammation, tissue regeneration and bacterial clearance [55,56]. In this context, understanding how materials could influence macrophage behavior and polarization is crucial for the transition from strategies to develop purely antibacterial materials to ones with immunomodulatory activity. In line with this transition, the aim of the present review was to identify and describe this new class of biomaterials in which the combination of antibacterial and immunomodulatory functions—particularly in terms of macrophage involvement—is employed to counteract, contain or prevent IAI in a preclinical in vivo setting.

A considerable heterogeneity has emerged in terms of types of materials used, functionalization strategy employed, and immune activation mechanisms, although the analysis of macrophage reprogramming results revealed that this is consistently associated with positive infection outcomes.

This interplay between antibacterial action and macrophage reprogramming is particularly evident in NIR responsive platforms. NIR responsive photothermal and photodynamic systems physically help kill bacteria by disrupting the integrity of cell membranes and organelles while modulating macrophage polarization toward anti-inflammatory yet bactericidal phenotype [7,17,21,22,25]. This created an osteoimmune environment favorable for the recruitment of bone marrow mesenchymal stem cells (BMSCs), extracellular matrix deposition and mineralization, and new bone formation [57,58,59]. Finally, as reported in the literature and in the study by Yu Y. et al., studies have shown that, compared to implantation with the material alone, stimulation with NIR drastically reduced the bacterial load. This was probably due to the material’s synergistic immunomodulatory and antibacterial action. At the molecular level, the ROS increase induced by the photodynamic effect of NIR led to a metabolic reprogramming in macrophages around the implant by regulating key signaling pathways, including Nf-kB, PI3K-Akt and MAPK [60,61,62,63].

This metabolic reprogramming is closely linked to immunometabolic adaptations that define macrophage functional phenotypes. Macrophage polarization is closely linked to metabolic reprogramming, which is a central feature of “immunometabolism.” During inflammatory activation, classically activated M1 macrophages primarily rely on glycolysis to meet their immediate energetic demands, a process driven in part by hypoxia-inducible factor-1α (HIF-1α), which promotes the expression of glycolytic enzymes and glucose transporters. Glycolytic activation is also linked to the pentose phosphate pathway, which produces the NADPH necessary for the generation of reactive oxygen species (ROS) via NADPH oxidase, thereby facilitating the killing of pathogens. By contrast, M2 macrophages are associated with tissue repair and the resolution of inflammation, relying predominantly on oxidative phosphorylation and fatty acid β-oxidation. Unlike the rapid, glycolysis-driven M1 response that characterizes the early inflammatory phase, M2 activation occurs during the resolution phase, when the immune response shifts progressively from clearing pathogens to restoring tissue. This longer-lasting functional state requires sustained ATP production, which is efficiently supported by oxidative metabolic pathways [64]. This metabolic profile enables prolonged cellular activity with reduced ROS generation, favoring anti-inflammatory and tissue-remodeling functions and reflecting adaptation to a resolving microenvironment. Within this metabolic framework, intracellular signaling pathways further refine macrophage polarization outcomes [65,66].

Notably, PI3K-Akt pathway is reported to play a context- and stage-dependent role in macrophage activation and polarization [67]. Although it generally promotes M2 polarization, different Akt isoforms (Akt1, Akt2, Akt3) finely determine the macrophage polarization fate [68]. During the early stages of inflammation, PI3K-Akt signaling contributes to fine-tuning rather than abolishing M1 responses, thereby preserving macrophage antimicrobial and bactericidal functions while preventing excessive activation through modulation of TLR/NF-kB signaling. In the later phase, this pathway contributes to the transition to the M2 phenotype and sustains anti-inflammatory programs [69].

Similarly, ultrasound-activated piezoelectric coatings have been shown to modulate macrophage polarization [37,43], accelerate bacterial killing and support tissue damage healing and implant integration in vitro and in IAI models [70,71]. Therefore, as with NIR-responsive biomaterials, ultrasound-activated piezoelectric platforms exert their effects through a combination of physical antibacterial actions and immune modulation. Even the release of metallic ions such as silver (Ag), copper (Cu), zinc (Zn) and strontium (Sr) from coated implants or nanomaterials could contribute to infection control through macrophage reprogramming, enhancing ROS generation [47,51] or regulating key signaling pathways in macrophages [31,33,41]. Together, these findings suggested that both externally triggered physical activation strategies and ion-releasing coatings could be effective approaches to enhance antimicrobial activity and support osteoimmune regulation, as consistently reported in the literature [72,73,74].

Interestingly, while titanium and PEEK implants displayed polarization toward either M1 or M2 macrophages depending on the specific functionalization strategy, nanomaterials predominantly promoted M1-type polarization. This divergence may reflect different therapeutic goals. M1-oriented responses are primarily aimed to enhance bacterial eradication through improvement of their bactericidal activity, whereas M2-skewing is more often associated with promoting tissue repair and implant integration after bacterial damage to tissues [75,76,77,78].

This difference can be attributed to the size of the materials. Materials within the phagocytosable range, such as most of investigated nanomaterials in the included studies, sustained inflammatory activation of macrophages compatible with pro-inflammatory M1 phenotype, while larger implants like titanium and PEEK were not phagocytosable and promoted tissue healing response associated more with M2 type [79,80,81]. For example, Cu nanodots embedded in mesoporous silicon nanoparticles (Motor@CP) [47], bovine serum albumin and manganese dioxide nanoclusters (BMCV) [48], and bismuth-based metal–organic frameworks (Bi-MOFs) [49], with sizes in the nanoscale range (114–808 nm), promoted a significant increase in the M1/M2 macrophage ratio. This effect was evidenced by the upregulation of M1 markers (CD86 and iNOS) and pro-inflammatory cytokines such as IL-6 and TNF-α. Conversely, larger biomaterials such as titanium and PEEK implants, functionalized with bioactive molecules (e.g., lactoferrin, antimicrobial peptide and quercetin) [31,34,40], nanoenzymatic systems such as glucose oxidase [32], or inorganic nanofilms [36], and characterized by millimetric dimensions, supported macrophage polarization toward the M2 phenotype. This was associated with increased expression of M2 markers (CD206 and ARG1) and anti-inflammatory mediators, including IL-10 and TGF-β.

Despite these differences, the included studies consistently reported favorable infection control outcomes, as with reduction in bacterial load and biofilm thickness, suggesting that multiple polarization approaches may converge toward effective antibacterial performances. Together, these findings suggest that modulation of macrophage behavior, orchestrated by material composition, surface and/or dimension properties, and exogenous stimulation strategies, can synergistically enhance both antimicrobial activity and tissue repair.

The results of macrophage-mediated infection control and tissue repair proposed by the included studies are promising, but several limitations should be acknowledged. All included studies used only rodent models that were predominantly male, excluding the use of large animal models like pigs [82], which more closely replicate human anatomy, immune responses [83], biomechanical conditions, and clinical implant dimensions [84,85]. The literature clearly documents that innate immunity is subject to sex-based differences: male rodents tend toward more pro-inflammatory responses, while females generally show a more regulatory profile, more efficient bacterial killing alongside better-regulated cytokine responses, and greater M2-like polarization capacity [86,87,88]. Within the included studies, only three employed female animal IAI models: Yamada et al. [20] and Chen C. et al. [50] both used mice for testing nanomaterials, while Xu K. [42] et al. investigated a functionalized titanium implant on a rat model. Despite using the same sex of animal model, these studies reported different outcomes in terms of polarization. Yamada et al. and Xu K. et al. observed a promotion towards the M1 phenotype, whereas Chen C. et al. observed M2 polarization. These results suggest that the type of material used and the experimental design are the main factors influencing polarization direction, rather than the sex of the animal itself. However, the predominant use of male animals in the remaining studies may introduce a systematic bias, so sex-stratified preclinical studies are required.

Furthermore, the assessment of biocompatibility and antimicrobial efficacy may be influenced by rodents’ rapid healing and lower immunological complexity [81,83,89,90]. These factors collectively limit the direct extrapolation of these preclinical findings to human clinical settings.

Moreover, heterogeneity in material types, functionalization strategies, infection induction protocols, implantation sites and quantitative, semi-quantitative or qualitative presentation of outcomes complicated the direct comparison among studies, precluding the performance of a metanalysis and limiting the synthesis to a narrative approach. Notably, eight of the twenty-four studies used subcutaneous implantation models to evaluate osteoimmunomodulatory and antibacterial materials, particularly for nanomaterials, [37,39,43,47,48,50,51,52]. While useful for preliminary biocompatibility assessment, subcutaneous implants do not replicate the bone microenvironment, which differs in immune cellular composition, vascularization and extracellular matrix and does not allow for osseointegration assessment [91,92,93]. The discussed differences should be considered when interpreting results from bone implants and subcutaneous models, especially in terms of macrophage behavior. Additionally, only the study of Chen B. et al. [36] investigated a maxillary implant site, and the reported results are not directly comparable to those obtained in bone implant models, given the distinct composition of the oral microflora, which comprises a highly diverse microbial community responsible for specific oral pathological conditions such as periodontitis and peri-implantitis, and which varies considerably across the different niches of the oral cavity, as well as the unique local immune response, shaped by continuous antigenic stimulation and specialized mucosal immunity mechanisms [94,95,96,97].

Although this review focused on immune modulation and infection control using next-gen biomaterials, it is the ultimate goal of biomaterials intended for orthopedic applications to achieve effective osseointegration. The investigated functionalized materials enhanced bone formation and peri-implant matrix deposition compared with unmodified controls such as pure PEEK or pure titanium [31,32,33,34,35,36,38,40,41,42,44,45,46]. This suggests that, beyond their antibacterial activity, these materials can support new bone deposition and stable integration, mitigating local inflammation through macrophage polarization toward the M2 phenotype and promoting tissue repair.

Nevertheless, methodological limitations should be considered. Most studies showed a high or unclear risk of bias, particularly regarding randomization procedures, allocation concealment, housing conditions, and blinding during surgical procedures and outcome assessment. These factors may have influenced the robustness and reproducibility of the reported results and could have contributed to an overestimation of treatment efficacy. Beyond methodological concerns, safety assessments were variably reported. While the most of studies (10/16, 62.5%) reported no histopathological abnormalities in major organs (liver, kidney, lungs, spleen and heart) or biochemical parameter alterations on routine blood tests, demonstrating overall biosafety, nanomaterials were less consistently evaluated [51,52]. As highlighted by Codispoti et al. [98], the safety of nanomaterials remains a critical aspect, despite their promising antimicrobial potential, due to the risk of unintended immune activation or chronic inflammatory responses [99,100,101,102]. In addition, toxicological investigations are required to exclude their accumulation in clearance organs such as the liver, spleen and kidney [103,104]. Thus, further studies would be valuable to better define the balance between biological safety, long-term tissue effects and efficacy in view of potential clinical translation.

Finally, it is clear that the immune response to IAIs cannot be attributed to the activity of a single cellular population, as considered in the present review. The immune system is highly complex, featuring dynamic and constantly evolving interactions among different cell types (such as macrophages, T and B lymphocytes, and neutrophils) and soluble mediators (cytokines, chemokines). This multifactorial network drives several scenarios, from chronic inflammation to implant integration or rejection, complicating the identification of therapeutic targets. Within this network, targeted approaches focusing on key pathways—such as macrophage TLR signaling or NF-κB-mediated inflammation—provide a rational starting point for therapeutic development and the testing of immunomodulatory strategies aimed at reducing biofilm burden and improving implant tolerance. In this context, myeloid-derived suppressor cells (MDSCs), a heterogeneous myeloid-derived cell population, have also been implicated in modulating macrophage function under chronic infection conditions [105,106,107]. During IAI, MDSCs could exert their immunosuppressive role on macrophages under the influence of S. aureus biofilm, driving a shift from pro-inflammatory M1 macrophages to the anti-inflammatory and pro-fibrotic M2 subtype, thereby supporting bacterial persistence and immune evasion [19,20,108,109,110,111,112,113,114].

These considerations highlight the need to use more advanced preclinical models in the future, given the complexity of the in vivo osteoimmune environment. This is crucial in order to determine whether the antibacterial efficacy of functionalized biomaterials involves not only direct modulation of macrophage behavior, but also their influence on other immune networks, orchestrating in turn macrophage phenotype.

## 5. Conclusions

The evidence discussed in this systematic review indicated that the successful management of *S. aureus* IAI relies not only on the intrinsic antibacterial properties of biomaterials, but also on their capacity to modulate macrophage polarization state. Both M1- and M2-oriented responses proved compatible with effective infection control, depending on material properties and functionalization strategies, sometimes combined with externally triggered stimuli. Despite relevant methodological limitations, macrophage-targeted immunomodulation might represent a promising approach to simultaneously controlling infection and supporting peri-implant tissue healing and implant osseointegration.

## Figures and Tables

**Table 1:** Full search strategies for database queries.

Database	Search Keywords	Search Period	Limits	Search Date
PubMed	(((Staphylococcus Aureus) OR (S. Aureus)) AND ((implant-associated infection) OR (periprosthetic infection) OR (peri-implant infection) OR (IAI) OR (peri-prosthetic joint infection) OR (PJI)) AND (macrophage)))	From 1 January 2015– 31 December 2025	English	4 November 2025
Web of Science	TS = (“staphylococcus aureus” OR “S. aureus”) AND TS=(“implant-associated infection” OR “periprosthetic infection” OR “peri-implant infection” OR “IAI” OR “periprosthetic joint infection” OR “PJI”) AND TS=(“macrophage”)	From 1 January 2015 –31 December 2025	English	4 November 2025
Scopus	(((TITLE-ABS-KEY (“staphylococcus aureus”) OR TITLE-ABS-KEY (“S. aureus”))) AND ((TITLE-ABS-KEY (“implant-associated infection”) OR TITLE-ABS-KEY (“periprosthetic infection”) OR TITLE-ABS-KEY (“peri-implant infection”) OR TITLE-ABS-KEY (“IAI”) OR TITLE-ABS-KEY (“peri-prosthetic joint infection”) OR TITLE-ABS-KEY (“PJI”))) AND (TITLE-ABS-KEY (“macrophage”)))	From 1 January 2015 –31 December 2025	English	4 November 2025
Embase	“Staphylococcus Aureus” OR “S. Aureus” AND “implant-associated infection” OR “periprosthetic infection” OR “peri-implant infection” OR “IAI” OR “peri-prosthetic joint infection” OR “PJI” AND “macrophage”	From 1 January 2015 –31 December 2025	English	4 November 2025

**Table 2:** Summary of the in vivo preclinical studies.

Tested Material (Size)	In Vivo IAI Model	IAI Model	Administration Time	Microbiological Investigations	Macrophage Reprogramming Assessment	Experimental Times	Results	Signaling Pathways	References
PEEK-PDA-Sr/AMP, NR	32 male SD rats, divided into 4 groups (*N*= 8): 1. PEEK 2. PEEK-DA 3. PEEK-DA-Sr 4. PEEK-PDA-Sr/AMP	Femur defect (1.2 × 10 mm) was filled with *S. aureus* (ATCC 25923) suspension (10^4^ CFU/mL) and then tested material was inserted	/	Histology: H&E and Giemsa stainings	IHC: CD86 and CD206	IHC: 4 weeks after IAI	Histology: No inflammatory reactions or bacterial colonies in bone of PEEK-PDA-Sr/AMP-treated rats vs. other groups IHC: Significantly ↑ CD206+ and ↓ CD86+ cells in PEEK-PDA-Sr/AMP vs. all other groups	Blocking of Nf-kB pathway by Sr ions	Wang L. 2025 [31]
SP/GOx/MnCO implant (2 d × 4 L mm)	30 male SD rats, 8–10 weeks old, divided into 5 groups (N = 6): 1. SPEEK 2. SP/GOx 3. SP/GOx + NIR 4. SP/GOx/MnCO 5. SP/GOx/MnCO + NIR	Cylindrical defects in tibial metaphysis, implant insertion and *S. aureus* (ATCC.25923) injected into the implant cavity	/	CFU count SEM IVIS	Immunofluorescence: Inos, CD206, IL-6 and TNF-α ELISA: IL-6 and TNF-α RT-Qpcr: Inos and CD206	CFU, SEM and IVIS: 3 d after IAI Immunofluorescence, ELISA and RT-qPCR: 1 w after IAI	CFU: SP/GOx/MnCO with NIR group has the lowest CFU count SEM: The SP/GOx/MnCO group has cell membrane rupture vs. intact SPEEK cells IVIS: DiR-labeled S. aureus radiant efficiency decreased drastically in SP/GOx/MnCO + NIR-treated rats Immunofluorescence: Significantly ↓ Inos+, IL-6+, TNF-α+ and ↑ CD206+ cells in SP/Gox/MnCo + NIR group vs. SPEEK ELISA: Significantly ↓ IL-6 and TNF-α in SP/Gox/MnCo + NIR group vs. SPEEK RT-Qpcr: Significantly ↓ Inos and ↑ CD206 expression in SP/Gox/MnCo + NIR group vs. SPEEK	Glucose-activated CO release suppresses MAPK-mediated pro-inflammatory signaling in macrophages, limits excessive M1 polarization, and promotes an inflammation resolving	Li J. 2024 [32]
STN-SF/ZIF-8@B (258.24 ± 39.77, average dimension of ZIF-8@B NPs)	9 adult male SD rats, divided into 3 groups (N = 3): 1. Ti 2. STN 3. STN-SF/ZIF-8@B	Femur cylindrical defect (1.8 mm d) filled with *S. aureus* suspension (NCTC 8325) (10^6^ CFU/mL) and implant insertion	/	CFU count	IHC: iNOS and CD206	CFU: 7 d after IAI IHC: 1 week after IAI	CFU: Significantly ↓ in STN-SF/ZIF-8@B-treated group vs. Ti group IHC: Significantly ↑ CD206+ and ↓ iNOS+ cells in STN-SF/ZIF-8@B vs. Ti group	M2 polarization promoted by STAT6 activation and Nf-kB inhibition through Zn2+	Tian Z. 2025 [33]
LF/Co-Mg-TN, coating on Ti surface (10 mm × 10 mm × 1 mm)	9 adult male SD rats, divided into 3 groups (N = 3): 1. Ti 2. Co-Mg-TN 3. LF/Co-Mg-TN	Femur cylindrical defect (1.8 mm d) filled with *S. aureus* suspension (NCTC8325) (10^6^ CFU/mL) and implant insertion	/	CFU count	IHC: iNOS and CD206	CFU: NR IHC: 1 week after IAI	CFU: NR IHC: Significantly ↑ CD206+ and ↓ iNOS+ cells in LF/Co-Mg-TN group vs. Ti group	/	Wang J. 2025 [34]
Gel@MX-ZIF8/CA, coating on Ti surfaces (10 mm × 10 mm × 1 mm)	9 adult male SD rats, divided into 3 groups (N = 3): 1. Ti 2. MX 3. Gel@MX-ZIF8/CA	Femur cylindrical defect (1.8 mm d) filled with *S. aureus* (NCTC8325) suspension (10^6^ CFU/mL) and implant insertion	/	CFU count	IHC: iNOS and CD206	CFU and IHC: 1 week after IAI	CFU: Significantly ↓ in Gel@MX-ZIF8/CA group vs. Ti group IHC: Significantly ↑ CD206+ and ↓ iNOS+ cells in Gel@MX-ZIF8/CA group vs. Ti group	/	Guan J. 2025 [35]
Ti-BTNT-ZnO nanofilm (d 71.0 ± 3.6 nm, l 2.0 ± 0.3 μm) on Ti surfaces	18 male SD rats, 3312 weeks old, divided into 3 groups (N = 6): 1. Ti 2. SLA 3. Ti-BTNT-ZnO	Maxilla implant after immersion into *S. aureus* (ATCC 6538), *P. gingivalis* (ATCC 33277) and *F. nucleatum* (ATCC 25586) suspension (10^7^ CFU/mL) and NIR after implantation (0.4 W cm^−2^ 808 nm NIR, 10 min). Same procedure for microbiological investigations, where the SLA group was substituted with Ti + minocycline and in total 9 rats were used (*n* = 3)	/	Roll-plate method	IHC: CD86 and ARG-1	Roll-plate method: 24 h after IAI IHC: 3 d and 1 week after implantation	Roll-plate method: Ti-BTNT-ZnO showed the fewest bacterial colonies and significant antibacterial efficiency IHC: Significantly ↑ Arg-1+ at 3 d and ↓ CD86+ cells both at 3 and 7 d in Ti-BTNT-ZnO group vs. Ti group	/	Chen B. 2024 [36]
BaTiO3-x/LA coating on Ti implant (1.5 × 3 mm)	Male Kunming rats, 8 weeks old, divided into 4 groups (*N* = 6): 1. Ti 2. Ti + US, 3. BaTiO3-x/LA, 4. BaTiO3-x/LA + US	The implant was immersed in MRSA bacterial solution (10^8^ CFU/mL) for 48 h, implanted into the back and irradiated by US (1.5 W/cm^2^, 1 MHz, 50% duty cycle) for 15 min	/	Antibacterial rate Histology: H&E and Giemsa on periimplant tissues	IHC: iNOS and CD206	Antibacterial rate and histology: 2 d after IAI IHC: 4 h and 72 h after IAI	Antibacterial rate: Significantly ↑ in BaTiO3-x/LA + US (97.54%) vs. the others Histology: ↓ immune cells and no bacteria in BaTiO3-x/LA + US group vs. Ti and BaTiO3-x/LA groups IHC: ↑ iNOS+ and ↓ CD206+ cells at 4 h in BaTiO3-x/LA + US group vs. all groups, ↑ CD206+ cells and -/- iNOS at 72 h in BaTiO3-x/LA + US group vs. Ti and Ti + US groups	ROS catalyze NO production by LA, stimulating M1 polarization	Sun M. 2024 [37]
CAR-MΦ gene-activating coating on Ti implant (10 L × 1 d mm)	C57BL/6J mice, divided into 5 groups: 1. CTR 2. Ti-PNP 3. TishRNA@PNP 4. Ti-CAR@PNP 5. Ti-pPNP	Intramedullary femoral canal filled with MRSA (ATCC43300) (10^5^ CFU/mL). Then CTR, Ti-PNP, TishRNA@PNP, Ti-CAR@PNP, or Ti-pPNP were implanted	/	CFU count SEM Histology: Gram staining	FC: CD80, CD206, CD86	CFU and FC: 1 week after IAI SEM and histology: 2 weeks after IAI	CFU: 25 mice of Ti-pPNP group showed no bacteria vs. 4 mice of CTR group; SEM and Gram staining: no viable planktonic bacteria in Ti-pPNP group vs. present biofilm aggregates into the others. No infection in 83% of mice in Ti-pPNP group FC: Significantly ↓ CD206+ (8.16%) and ↑ CD80 + (31.5%) and CD86+ (7.29%) cells in Ti-pPNP group vs. CTR	Promoting enhanced recognition of S. Aureus by macrophages coding CAR and improving their bactericidal activity through CASP11 Mrna silencing	Li Z. 2023 [38]
TNT@IL-4/GelMA@CaO2 (NR)	Male Balb/c mice, 6 weeks old, divided into 4 groups: 1. Ti 2. TNT@IL-4 3. GelMA@CaO2 4. TNT@IL-4/GelMA@CaO2	Subcutaneous implantation of two samples/group and injection of *S. aureus* (10^5^ CFU/mL)	/	CFU count Histology: Giemsa staining	IHC: TGF-β, iNOS, IL-10 and TNF-α Immunofluorescence: CD86 and CD206	CFU and histology: 2 d after IAI IHC and immunofluorescence: 7 d after IAI	CFU: Significantly ↓ CFU in GelMA@CaO2 and TNT@IL-4/GelMA@CaO2 groups vs. Ti and TNT@IL-4 groups Giemsa staining: Reduced neutrophil infiltration in all groups vs. Ti group IHC: Significantly ↓ iNOS + and TNF-α+ and ↑ TGF-β+ ND IL-10+ cells in TNT@IL-4/GelMA@CaO2 group vs. TNT@IL-4 Immunofluorescence: Significantly ↓ CD86+ and ↑ CD206+ cells in TNT@IL-4/GelMA@CaO2 group vs. TNT@IL-4	ROS production induced by CaO2 NPs and IL-4 releasing through the implant promote polarization toward M2-type macrophages in order to create an anti-inflammatory and pro-reparative microenvironment	He Y. 2021 [39]
SQPdFT (15 d × 1 L mm)	30 male SD rats, 8 weeks old, divided into 6 groups (N = 5): 1. CTR (Ti) 2. Ti + MRSA 3. 3DFT + MRSA 4. PdFT + MRSA 5. SPdFT + MRSA 6. SQPdFT + MRSA	Ti implants exposed to MRSA (ATCC 6538) (10^6^ CFU/mL) and implanted into the distal femur	/	Antibacterial rate Histology: Gram and H&E stainings	IHC: IL-1β, IL-6, IL-10 and TNF-α Immunofluorescence: CD206 and Arg-1, CCR7 and CD86	Antibacterial rate, histology, IHC and immunofluorescence: 6 w after IAI	Antibacterial rate: Significantly ↑ in SQPdFT group vs. SPdFT group Histology: Severe bacterial infection around the implants and inflammatory infiltrate in all groups except SQPdFT IHC: Significantly ↓ IL-1β, IL-6, TNF-α expression and ↑ IL-10 in SQPdFT + MRSA group vs. Ti + MRSA group Immunofluorescence: Significantly ↑ CD206+ and Arg-1+ and ↓ CCR7+ and CD206+ cells in SQPdFT + MRSA group vs. Ti + MRSA group	SQPdFT decreases ROS and inflammatory factor production, activating PPARγ/NF-κB signaling pathway and promoting M2 polarization	Yang N. 2024 [40]
PGA/Ag platform (NR)	36 male SD rats, divided into 3 groups (N = 6): 1. SLA 2. PGA 3. PGA/Ag	Two implants per animal inserted into each tibial hole in bone marrow cavity, injected with *S. aureus* suspension (10^4^ CFU/mL) (ATCC25923)	/	CFU count	IHC: iNOS and Arg-1	CFU: 3 d after IAI IHC: 4 w and 8 w after IAI	CFU: Significantly ↓ CFU in PGA/Ag group (90%) vs. the others IHC: ↑ Arg-1+ and ↓ Inos + cells in PGA/Ag group vs. SLA and PGA groups	γ-PGA slowly releases Ag+ in responses to pH variations in IAI microenvironment and reduces inflammation	Yu Z. 2025 [41]
Ti-TF-acBSP implant (NR)	Female SD rats, divided into 6 groups: 1. Ti 2. Ti + NIR 3. Ti-Tf 4. Ti-Tf + NIR 5. Ti-Tf-acBSP 6. Ti-Tf-acBSP + NIR	Femur cylindrical defect (1.2 mm d) filled with *S. aureus* suspension (ATCC29213) (10^6^ CFU/mL) and implant insertion and irradiation with NIR	/	CFU count Histology: Giemsa and H&E stainings	IHC: CD86	CFU and histology: 7 d after IAI IHC: 4 d, 7 d, 14 d after IAI	CFU: Significantly ↓ CFU in Ti-Tf-acBSP group w/ and w/o NIR vs. Ti group Histology: Few infiltrating cells and bacteria in the Ti-TF-acBSP+NIR group vs. other groups IHC: Significantly ↑ CD86 expression in Ti-TF-acBSP and TiTF-acBSP+NIR groups at all time points compared to Ti and Ti + NIR groups, where the marker was not found	Fe(III)-TA NPs promote photothermal therapy induced by NIR and BSP activates M1-type macrophages thank to their high affinity for TLRs	Xu K. 2023 [42]
PiezoTi (NR)	Male Wistar rats, 6 weeks old, divided into 5 groups: 1. CTR (pristineTi w/o *S. aureus*) 2. Ti 3. piezoTi 4. Ti + US 5. piezoTi + US	Subcutaneous implantation of *S. aureus* (10^7^ CFU/mL) (ATCC6538) biofilm-contaminated Ti and piezoTI pillars	/	CFU count Histology: H&E staining	RT-qPCR: TNF-α, IL-1β, IL-6 and Inos Immunofluorescence: CD11b and CD86	CFU: 24 h after US treatment Histology and RT-qPCR: 4 h, 12 h and 96 h after IAI Immunofluorescence: 4 h and 96 h after IAI	CFU: Significantly ↓ CFU in piezoTi + US vs. Ti Histology: At 96 h, serious inflammatory responses were observed for Ti, Ti+US, and piezoTi groups, whereas a mild inflammatory response was revealed in piezoTi+US group, thanks to bacterial clearance RT-qPCR: Significantly ↓ TNF-α at 96 h in piezoTi + US group compared to the others, significantly ↓ IL-1β in CTR and piezo-Ti + US groups vs. Ti and piezoTi, significantly ↓ IL-6 in CTR and piezo-Ti + US groups vs. Ti and Ti + US, significantly ↓ Inos in CTR and piezoTi + US groups vs. others Immunofluorescence: ↑ CD86+ cells at 4 h and ↓ at 96 h in piezoTi + US group vs. others	PiezoTi, activated by US, promotes ROS production to piezodynamically kill S. Aureus and activates macrophages through PI3K-AKT and MAPK pathway and is able to phagocyte bacteria	Li K. 2023 [43]
3DMA (TiO2 NT and PVDF) on Ti surface (NR)	Male SD rats, 10 weeks old, divided into 2 groups (*n* = 3 per group): 1. PT 2. 3DMA	Implantation of *S. aureus* (10^7^ CFU/mL) (ATCC 25 923) biofilm-contaminated PT and 3DMA samples in distal femoral defect	/	Histology: H&E and Giemsa stainings	Immunofluorescence: Inos and CD206	Histology and immunofluorescence: 3 d after IAI	Histology: 3DMA group exhibited fewer inflammatory reactions around the infected bone than the PT group Immunofluorescence: Significantly ↑ CD206+ and ↓ Inos+ cells in 3DMA-treated group vs. PT group	Piezoelectric self-generated electrical stimulation modulates macrophage membrane polarization, downregulating M1 and promoting M2 anti-inflammatory phenotype	Sun L. 2023 [44]
Vaterite(Van)-Alg/Lipo(IL-12)-Vaterite(Van) coating on Ti implant	36 male SD rats, divided into 5 groups: 1. Blank 2. Vaterite(Van) 3. Vaterite(Van)-Alg 4. Vaterite(Van)-Alg/Lipo(IL-12) 5. Vaterite(Van)-Alg/Lipo(IL-12)- Vaterite(Van)	Tibial plateau defect filled with MRSA (ATCC 43300) and implantation of TI screw with different coatings into the channel. After 2 w, another tibial channel created and injected with MRSA, and then a hole into the bone marrow was created and injected with 5% sodium morrhuate and S. aureus suspension (10^6^ CFU/mL). Same procedure at 4 w	/	CFU count	IHC: CD68, Inos, CCR7	CFU: 4 w after IAI IHC: 2 w, 4 w and 6 w after IAI	CFU: Significantly ↓ CFU in Vaterite(Van)-Alg/Lipo(IL-12) and Vaterite(VanAlg/Lipo(IL-12)-Vaterite(Van) vs. the other groups IHC: Significantly ↑ CCR7, CD68 and Inos expression at 14 d and 28 d in Vaterite(Van)-Alg/Lipo(IL-12) and Vaterite(Van)-Alg/Lipo(IL-12)-Vaterite(Van) groups compared to the others	Sustained IL-12 release activates IL-12R–STAT4 and IFN-γ–STAT1 signaling in macrophages, inducing early M1 polarization (CCR7^+^, iNOS^+^) and suppressing M2	Lian Q. 2022 [45]
Ti-PDA@SNP-OGP (NR)	40 male SD rats, divided into 4 groups: 1. Ti 2. Ti + NIR 3. Ti-PDA@SNP-OGP 4. Ti-PDA@SNP-OGP + NIR	Femur cylindrical defect filled with Ti implants, after pre-formed *S. aureus* (10^7^ CFU/mL) biofilm, and irradiated by NIR	/	CFU count	ELISA: TNF-α and IL-6, TGF-β and IL-10 IHC: CD86 and CD206	CFU and IHC: 3 d after implantation	CFU: 6.2% of MRSA biofilms were eliminated without NIR irradiation vs. 95.7% on Ti-PDA@SNP-OGP ELISA: Significantly ↓ TNF-α and IL-6 in Ti-PDA@SNP-OGP groups vs. others; significantly ↑ TGF-β and IL-10 in Ti-PDA@SNP-OG group vs. Ti and Ti + NIR IHC: ↓ CD86 and ↑ CD206 in Ti-PDA@SNP-OGP + NIR group vs. Ti	Ti-PDA@SNP-OGP under NIR induces NO-mediated NF-κB inhibition and redox regulation, promoting macrophage polarization toward the M2 phenotype	Yu Y.L. 2023 [46]
Motor@CP nanomotor (808 nm)	Male Balb/C mice, 8 weeks old, divided into 5 groups: 1. Control 2. CP 3. Motor 4. Motor@CP 5. Motor@CP + NIR	Subcutaneous PEEK implantation after pre-formed *S. aureus* (MRSA, ATCC 43300) biofilm (bacterial suspension, 10^6^ CFU/mL) on the back of the animal’s neck	Day 2 after surgery, different treatments were injected	CFU count SEM	ELISA on peripheral tissue homogenates: TNF-α, IL-6, IL-10, VEGF FC on IDLNs: CD86 and CD206 IHC: TNF-α and iNOS	CFU, SEM and ELISA: at day 10 after IAI FC and IHC: at day 5 after IAI	CFU count: Significant ↓ of CFU in Motor@CP + NIR group vs. the others SEM: ↓ viable cells after Motor@CP + NIR treatment ELISA: Significantly ↑ TNF-α and IL-6 and ↓ IL-10 and VEGF in Motor@CP group vs. CTR FC: Significantly ↑ CD86+ cells and ↓ CD206+ ones after Motor@CP treatment vs. CTR IHC: ↓ TNF-α and iNOS in CP, Motor@CP and Motor@CP + NIR groups vs. Motor and CTR ones	ROS production by Cu ions catalysis	He L. 2025 [47]
BMCV NPs (114 nm)	Male Balb/c mice, 8 weeks old, divided into 4 groups: 1. CTR 2. Ce6 3. BMC 4. BMCV	Subcutaneous implantation of Ti sheet (1 cm) on the animal’s back and injection of luminescent *S. aureus* ST8-lux suspension on the Ti surface. After 28 days, implants were removed and substituted with new implant to create a recurrent IAI model	Day 1 after surgery, NP injection and laser (660 nm) treatment (PDT) were performed for 15 min	Infection area measurement CFU count CLSM SEM Histology: HE and Giemsa	Immunofluorescence: iNOS and CD206 ELISA: IL-10 and TNF-α	Infection area measurement: at days 0, 2, 4, 6, 8, 10, and 12 after treatment CFU, CLSM, SEM and histology: 14 d after treatment Immunofluorescence: 4 d and 14 d after treatment	Infection area measurement: No signs of infection with healthy skin after BMCV NP treatment vs. the others CFU: Significantly ↓ in BMCV-treated groups vs. other treated groups CLSM and SEM: dense and thick structure of the *S. aureus* biofilm in CTR, and scattered dead cells in the BMCV-treated group Histology: Milder inflammatory response with fewer residual bacteria in the BMCV group vs. significant neutrophil infiltration and bacteria in the Ce6 group Immunofluorescence: ↓ CD206+ and ↑ Inos+ at 4 d and ↓ Inos+ and ↑ CD206+ at 14 d after BMCV treatment vs. CTR ELISA: At 4 d significantly ↑ TNF-α and ↓ IL-10, while at 14 d significantly ↓ TNF-α and ↑ IL-10 in BMCV group vs. CTR Immunofluorescence: ↑ CD206+ at 14 d after BMCV treatment in the recurrent IAI model	Light-induced ROS production and immunity activation by releasing immune costimulatory factors from bacterial cells death	Jiang F. 2024 [48]
Oligomycin NPs (CTO, 80 nm)	Male and female C57BL/6NCrl mice, 8 weeks old, divided into 4 groups: 1. Oligo 2. CT 3. CT + Oligo 4. CTO	Femoral intercondylar notch and an orthopedic-grade Kirschner wire (0.6 mm d, Nitinol) implanted, leaving ~1 mm protruding into the joint space for exposure to *S. aureus* USA300 LAC13c (10^3^ CFU/mL)	Single i.a. injection of oligomycin NPs at either day 3 or 7 post IAI	CFU count on implant surface, knee joint, periimplant tissue and femur	RT-qPCR: Arg-1, IL-10, Trem2, HIF-1α, iNOS, TNF-α, Trem1	CFU: Day 7, 14, 21, 28 after IAI RT-qPCR: 10 d after IAI	CFU: Significantly ↓ CFU in all tissues after CTO treatment compared to others RT-qPCR: ↑ HIF-1α, iNOS, TNF-α, Trem1 an ↓ Arg-1, IL-10, Trem2 after CTO treatment vs. CT	Oligomycin inhibits OxPhos to metabolically reprogram macrophages toward pro-inflammatory phenotype for biofilm clearance	Yamada K.J. 2020 [20]
Bi-MOF (600 nm)	Male C57BL/6 mice, 12 weeks old, divided into 5 groups: 1. CTR (PBS injection) 2. IAI + PBS 3. IAI + Bi-MOF 4. IAI + Clo 5. Bi-MOF + Clo	Stainless-steel pin (0.3 × 3 mm) inserted into a femural hole and *S. aureus* (IVISbrite 119240)(10^6^ CFU/mL) injected	Day 1 after surgery, Bi-MOF or PBS was injected into the femural defect	IVIS; CFU count	FC: CD206, CD86 and M1/M2 ratio	IVIS: Every 3 d after day 1 post surgery until day 12 CFU: Day 12 after treatments FC: 6 d after IAI (on bone marrow)	IVIS and CFU: Bi-MOF decreased the bacterial load (ns) FC: ↑ CD86+ and significantly ↑ M1/M2 ratio, ↓ CD206+ cells after IAI + Bi-MOF treatments compared to IAI + PBS	Bi-MOF acts as an intracellular H2S scavenger that enhances the antibacterial response of macrophages by inhibiting HIF-1α S-sulfhydration and subsequent degradation	Yang Y. 2024 [49]
ICG+RSG-DPPC NPs (150–250 nm)	Female C57BL/6 mice, 8 weeks old, divided into 4 groups: 1. PBS + NIR 2. RSG + NIR 3. ICG + NIR 4. ICG + RSG + NIR	Subcutaneous implantation of PVA hydrogels loaded with different NPs, pre-immersed into the bacterial suspension (ATCC 25923), then irradiated with NIR	/	NR	FC: CD206	FC: 3 d after implantation	FC: Significantly ↑ CD206+ cells in ICG + RSG + NIR group (55%) vs. PBS + NIR (30.9%) group	ICG enhances antibacterial effect of NIR therapy, while RSG is a PPAR-γ agonist, promoting polarization in the M2-type direction	Chen C. 2023 [50]
MACG (NR)	BALB/c mice, divided into 6 groups: 1. CTR 2. NIR 3. MACG + DMTU 4. MACG 5. MACG + DMTU + NIR 6. MACG + NIR	Subcutaneous implantation of PEEK washer, after culturing with MRSA (ATCC 43300) (bacterial suspension 10^7^ CFU/mL)	Day 1 after implant, different treatments were injected and irradiated	CFU count on PEEK and peripheral tissue Histology: H&E and Giemsa stainings	Immunofluorescence: iNOS and CD206, TNF-α and IL-6	CFU, histology and immunofluorescence: 2 w after IAI	CFU: MACG + NIR group had the best biofilm elimination outcome Histology: No bacteria and the lowest inflammatory infiltrate in MACG + NIR group Immunofluorescence: ↑ Inos+ cells, TNF-α and IL-6 expressions and ↓ CD206+ cells in MACG + DMTU + NIR and MACG + NIR groups vs. MACG + DMTU and MACG	MACG promotes M1 macrophage polarization by releasing Cu ions and generating ROS in the acidic, H_2_O_2_-rich infection microenvironment	Su Z. 2023 [51]
MPS-PVP (3 h × 120–240 L nm)	40 male BALB/c mice, 6–8 weeks old, divided into 4 groups: 1. CTR 2. VAN 3. MPS1 (10 mg/kg) 4. MPS2 (20 mg/kg)	Subcutaneous implantation of Ti plate (6 mm d) and MRSA injection (ATCC43300, 10^6^ CFU/mL) onto the implants	Day 3 after implant, different treatments were injected	CFU count on implant and peripheral tissue SEM	FC on IDLNs: CD80+CD206- and CD80+CD86+ ELISA: TNF-α, IL-6, IL-1β and IL-12	CFU, SEM, FC and ELISA: 10 d after IAI	CFU: Significantly ↓ CFU in MPS2-treated group vs. MPS1-treated group SEM: Biofilm eradication after MPS2 treatment FC: ↑ CD80+ cells after MPS-PVP treatment ELISA: significantly ↑ TNF-α, IL-6, IL-1β and IL-12 expression after MPS-PVP treatment	MnPSe3 nanosheet (MPS NS) can act as an inorganic immune-stimulatory agent, promoting M1 polarization and damaging biofilm by ROS production	Yang C. 2022 [52]

Abbreviations: **Motor@CP** = copper peroxide (CP) nanodots encapsulated into mesoporous silicon nanoparticle (MSN); **IAI** = implant-associated infection; **NIR** = near-infrared; **SEM** = scanning electron microscopy; **ELISA** = Enzyme-Linked Immunosorbent Assay; **FC** = flow cytometry; **CFU** = colony-forming unit; **IDLNs** = infected-draining lymph nodes; **IL-6** = interleukin-6; **IL-10** = interleukin-10; **TNF-α** = tumor necrosis factor-α; **VEGF** = vascular and endothelial growth factor; **iNOS** = inducible nitric oxide synthetase; **CTR** = control; **CD** = cluster of differentiation; **↑** = increase; **↓** = decrease; **ROS** = reactive oxygen species; **Cu** = copper; **K** = Kirschner; **d** = diameter; **PEEK** = polyetheretherketone; **MRSA** = Methicillin-resistant Staphylococcus Aureus; **PEEK-PDA-Sr/AMP **= PEEK–polydopamine (PDA)-chelating strontium; (Sr) and immobilizing antimicrobial peptide PMAP-36; **SD** = Sprague–Dawley; **IHC** = immunohistochemistry; **H&E** = hematoxylin and eosin staining; **NF-kB** = nuclear factor kappa-light-chain-enhancer of activated B cells; **NR** = not reported; **STN-SF/ZIF-8@B** = metal–organic framework encapsulating bacitracin (ZIF-8@B) incorporated into strontium-doped titanium dioxide nanorod (STN) structure with silk protein (SF); **Ti** = titanium; **Zn** = zinc; **STAT6** = Signal Transducer and Activator of Transcription 6; **M1** = macrophage phenotype 1; **M2** = macrophage phenotype 2; **LF/Co-Mg-TN** = amyloid lactoferrin (LF) loaded into cobalt- and magnesium-containing double hydroxides (Co-Mg) on titanium dioxide nano-net arrays (TNs); **Gel@MX-ZIF8/CA** = gelatin methacrylate (GelMA) hydrogel embedding TN nanotubes, coated by MXene and ZIF-8, for cinnamaldehyde (CA) release; **SLA** = self-limiting alcohol etching; **Ti-BTNT-ZnO** = titanium substrate decorated with zinc oxide deposited on black oxygen-deficient titanium dioxide nanotubes; **l **= length; **Arg-1** = arginase 1; **BaTiO3-x/LA** = oxygen-deficient barium titanate (BaTiO3) nanorod arrays and L-arginine (LA); **US** = ultrasound; **NO** = nitric oxide; **BMCV NPs** (**BSA@MnO2@Ce6@Van**) = BSA-MnO2 nanocluster loaded with the photosensitizer Ce6 and grafted vancomycin moieties onto the nanocluster surface via conjugation; **NP** = nanoparticle; **CLSM** = Confocal Laser Scanning Microscopy; **PDT** = photodynamic therapy; **MΦ** = macrophage; **CAR** = Chimeric Antigen Receptor; **CASP11** = caspase 11; **PNP** = peptide nanoparticle; **shRNA** = short hairpin RNA; **Ppnp** = pDNA-laden PNP; **mRNA** = RNA messenger; **TNT@IL-4/GelMA@CaO2** = titanium dioxide nanotubes (TNTs) loaded with interleukin-4 (IL-4) and coated with a gelatin methacryloyl hydrogel (GelMA)–calcium peroxide composite; **TGF-β** = transforming growth factor β; **Trem** = triggering receptor expressed on myeloid cells (1 or 2); **HIF-1α** = hypoxia-inducible factor-alpha; **OxPhos** = oxidative phosphorylation; **CTO** = Cy5/tuftsin/oligomycin; **Oligo** = oligomycin only; **CT** = empty nanoparticles; **RT-Qpcr** = Real Time-quantitative Polymerase Chain Reaction; **SQPdFT** = Silver–Quercetin/Polydopamine on Ti-based implant; **3DFT** = alkali-heat-treated Ti surfaces; **IL-1β** = interleukin-1β; **(PPARγ)/NF-κB** = peroxisome proliferator-activated receptor gamma/nuclear factor kappa-B; **PGA/Ag** = Ti nanotubes linked to poly-γ-glutamic acid (γ-PGA) incorporating silver ions (Ag+); **SP/GOx/MnCO** = sulfonated poly(etheretherketone)/glucose oxidase/manganese carbonyl nanocrystals; **NIR** = near infrared; **IVIS** = in vivo imaging system; **CO** = carbon monoxide; **Ti-TF-acBSP** = Fe(III) tannic acid (TA) nanoparticles and acetyl Bletilla striata polysaccharide on Ti surface; **TLRs **= Toll-like receptors; **piezoTi** = piezotronic Ti; **PI3K-Akt** = phosphoinositide 3-kinase/protein kinase B signaling pathway; **MAPK** = mitogen-activated protein kinase signaling pathway; **3DMA** = 3D multifunctional architecture; **NT** = nanotubes; **PVDF** = polyvinylidene fluoride: **PT** = pure Ti; **Vaterite(Van)-Alg/Lipo(IL-12)-Vaterite(Van)** = vancomycin-loaded vaterite/alginate-encapsulated IL-12 liposome/vancomycin-loaded vaterite; **IL-12** = interleukin 12; **CCR7** = C-C chemokine receptor type 7; **IL-12R-STAT4** = Interleukin-12 Receptor–Signal Transducer and Activator of Transcription 4; **IFN-γ-STAT1** = Interferon–gamma–Signal Transducer and Activator of Transcription 1; **Bi-MOF** = bismuth-based metal organic frameworks; **PBS** = phosphate buffer saline; **Clo** = clodronate; **H2S** = hydrogen sulfide; **Ti-PDA@SNP-OGP** = Ti functionalized with exoporous polydopamine nanoparticles loaded with the nitric oxide donor sodium nitroprusside and osteogenic growth peptide; **ICG+RSG-DPPC** = indocyanine green + rosiglitazone delivered by 1,2-dipalmitoylsnglycero-3-phosphocholine; **MACG** = biofilm microenvironment (BME)-responsive double-layered metal–organic framework (MOF) bionanocatalysts (MIL-100-2,2′-azino-bis (3-ethylbenzothiazoline-6-sulfonic acid) (ABTS)@CuBTC-glucose oxidase (Gox); **DMTU** = dimethylthiourea; **H2O2** = hydrogen peroxide; **MPS-PVP** = two-dimensional manganese chalcogenophosphate MnPSe3 nanosheets modified with polyvinylpyrrolidone; **h** = height; **VAN** = vancomycin; **ns** = not significant; / = No administration time.

## Data Availability

No new data were created or analyzed in this study. Data sharing is not applicable to this article.

## Supplementary Materials

The following supporting information can be downloaded at: https://www.mdpi.com/article/10.3390/jfb17070342/s1. PRISMA 2020 Checklist.
